# Case Report: Immune Checkpoint Inhibitors as a Single Agent in the Treatment of Metastatic Cervical Cancer

**DOI:** 10.3389/fonc.2022.856944

**Published:** 2022-04-06

**Authors:** Manasa Anipindi, Ryan J. Smith, Madiha Gilani

**Affiliations:** ^1^ Internal Medicine residency, Einstein Medical Center Montgomery, East Norriton, PA, United States; ^2^ Department of Radiology, Einstein Medical Center Montgomery, East Norriton, PA, United States; ^3^ Department of Oncology, Einstein Medical Center Montgomery, East Norriton, PA, United States

**Keywords:** metastatic cervical cancer, immune checkpoint inhibitors, pembrolizumab, PD-L1, single-agent immunotherapy

## Abstract

The incidence of cervical cancer has decreased in recent years due to widespread vaccination and routine screenings. It can be treated successfully, and the prognosis is also excellent if detected early. However, the 5-year survival rate for patients with stage IV cervical cancer is only 17% even with aggressive systemic chemotherapy. With the Food and Drug Administration (FDA)’s approval of immunotherapy, the prognosis has improved. We present a patient with stage IV cervical cancer who could not tolerate platinum-based chemotherapy and bevacizumab, so she was started on an immune checkpoint inhibitor, as her tumor was 100% programmed cell death ligand-1 (PD-L1) positive. She survived more than 2 years since the diagnosis of stage IV cervical cancer without any significant side effects. Based on our patient’s response, the use of immune checkpoint inhibitors as a single agent needs further research and probably can be considered in patients with stage 4 cervical cancer who cannot tolerate standard chemotherapy.

## Introduction

Cervical cancer remains a common cancer worldwide despite widespread preventive strategies (1). The American Cancer Society 2021 estimates there are about 14,480 new cervical cancer cases, out of which about 4,290 women will die, averaging about 11 deaths every day (2, 3). Prognosis remains poor even with the addition of bevacizumab to cytotoxic chemotherapy in patients with metastatic cervical cancer. Bevacizumab improves overall median survival by 3.7 months and was associated with severe side effects (4). The response to chemotherapy is usually limited, as most patients with advanced cervical cancers have a very brief response, and patients cannot receive multiple agents due to adverse side effects. Also, there is no evidence suggesting an increase in disease-free progression rate or overall survival in patients treated with second-line chemotherapy (5). The discovery of immune checkpoint inhibitors (ICIs) has changed the field of oncology over the past few years. The addition of programmed cell death protein-1 (PD-1) inhibitors to the treatment has further improved survival in metastatic cervical cancer patients (6, 7). Our patient has progressed to stage IV within a few months of her initial stage IB2 cervical cancer diagnosis. She received 6 cycles of weekly cisplatin with pelvic external beam radiation therapy and brachytherapy for her initial diagnosis of stage IB2 cervical cancer. Then she proceeded with chemotherapy for stage IV cervical cancer after lung metastasis, but she could not tolerate cisplatin-based chemotherapy or bevacizumab due to poor bone marrow reserve and renal issues. She was started on pembrolizumab, as her tumor was 100% programmed cell death ligand-1 (PD-L1) positive. She has been disease-free for more than 2 years and is continuing to maintain that response without any significant side effects.

## Case Discussion

**Figure 1:**
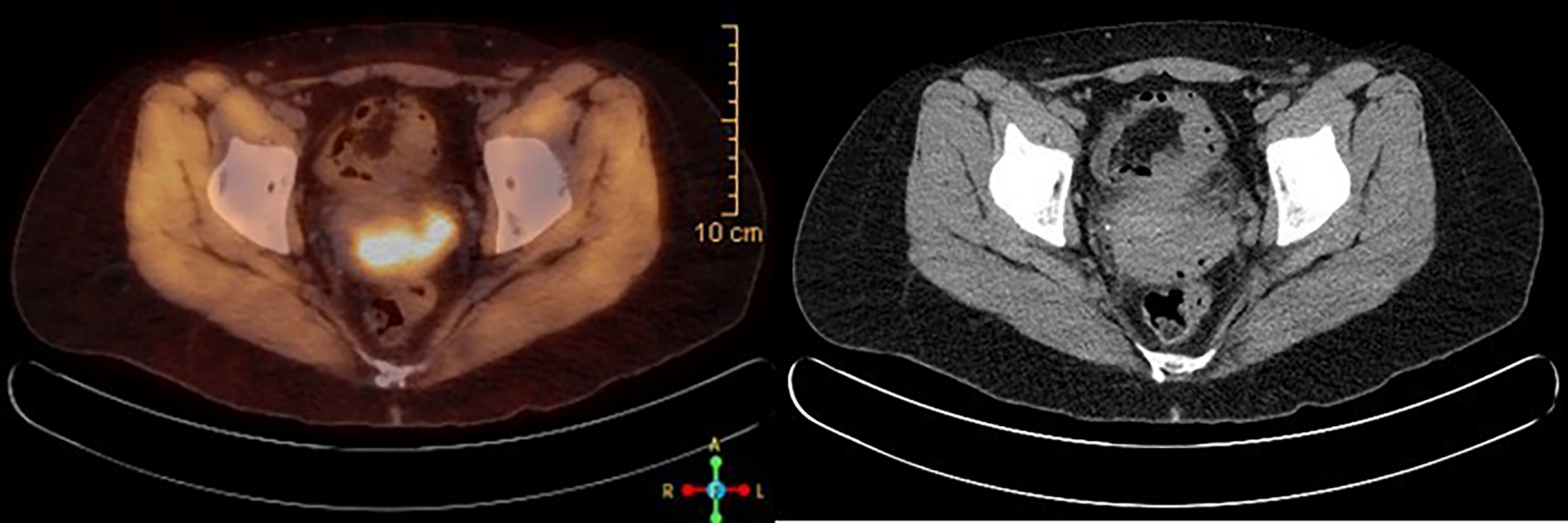
PET/CT scan at the time of diagnosis, showing FDG avid 4.4 × 4.8 × 2.7 cm cervical mass, consistent with known cervical cancer.

A 39-year-old female with a past medical history of anxiety, recurrent bacterial vaginosis, atypical squamous cells of undetermined significance (ASCUS) at age 28 status post loop electrosurgical excision procedure (s/p LEEP) with biopsy negative for cervical intraepithelial lesion or malignancy, and tobacco abuse went to her obstetrician–gynecologist (OB/GYN) for the complaints of vaginal odor and postcoital bleeding. She also reported unintentional weight loss of 18 pounds in the past 6 months. She had recurrent urinary tract infections and bacterial vaginosis diagnoses earlier that year that were successfully treated each time. Since the diagnosis of ASCUS, she had one human papillomavirus (HPV)/pap co-test negative at age 34. Her physical examination at the office showed a friable cervix with bleeding and discharge. She was treated for bacterial vaginosis infection first and was reexamined a week later after the infection has cleared. Reexamination under anesthesia revealed a cervical mass, so cystoscopy and proctoscopy with cervical biopsy were done. The results demonstrated a moderate to poorly differentiated invasive squamous cell carcinoma. She was diagnosed with stage I B2 cervical cancer in late 2018 after the biopsy and was started on chemoradiation. She received cisplatin weekly for six doses with a cumulative dose of 55.8 Gray of pelvic external beam radiation therapy, and suspicious lymph nodes were boosted. This was followed by five fractions of 3,000 centi-gray cervix 3-D image brachytherapy. Pretreatment MRI of the pelvis with and without contrast demonstrated a 4.4 × 4.8 × 2.7 cm of cervical mass with disruption of outer contour in the left aspect of the cervix. PET/CT demonstrated FDG activity in cervical 4.4 × 4.8 × 2.7 cm mass (**Figure 1**). Repeat MRI 2 months later showed interval and decrease in the size of cervical mass with small sub-centimeter bilateral internal iliac lymph nodes. She required frequent blood transfusions for anemia in this process.

**Figure 2:**
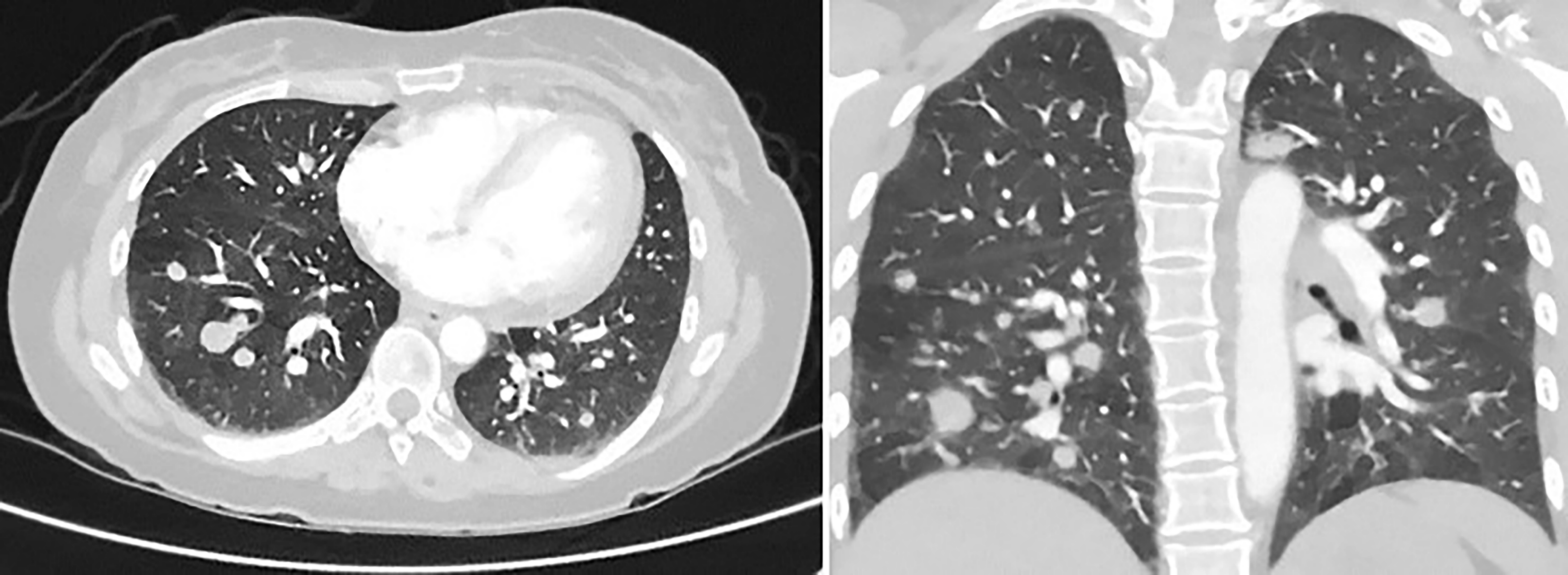
CT chest with contrast, 5 months later, revealing multiple pulmonary parenchymal nodules of varying sizes throughout the lungs, consistent with diffuse pulmonary metastasis. It also showed mediastinal and hilar adenopathy.

**Figure 3:**
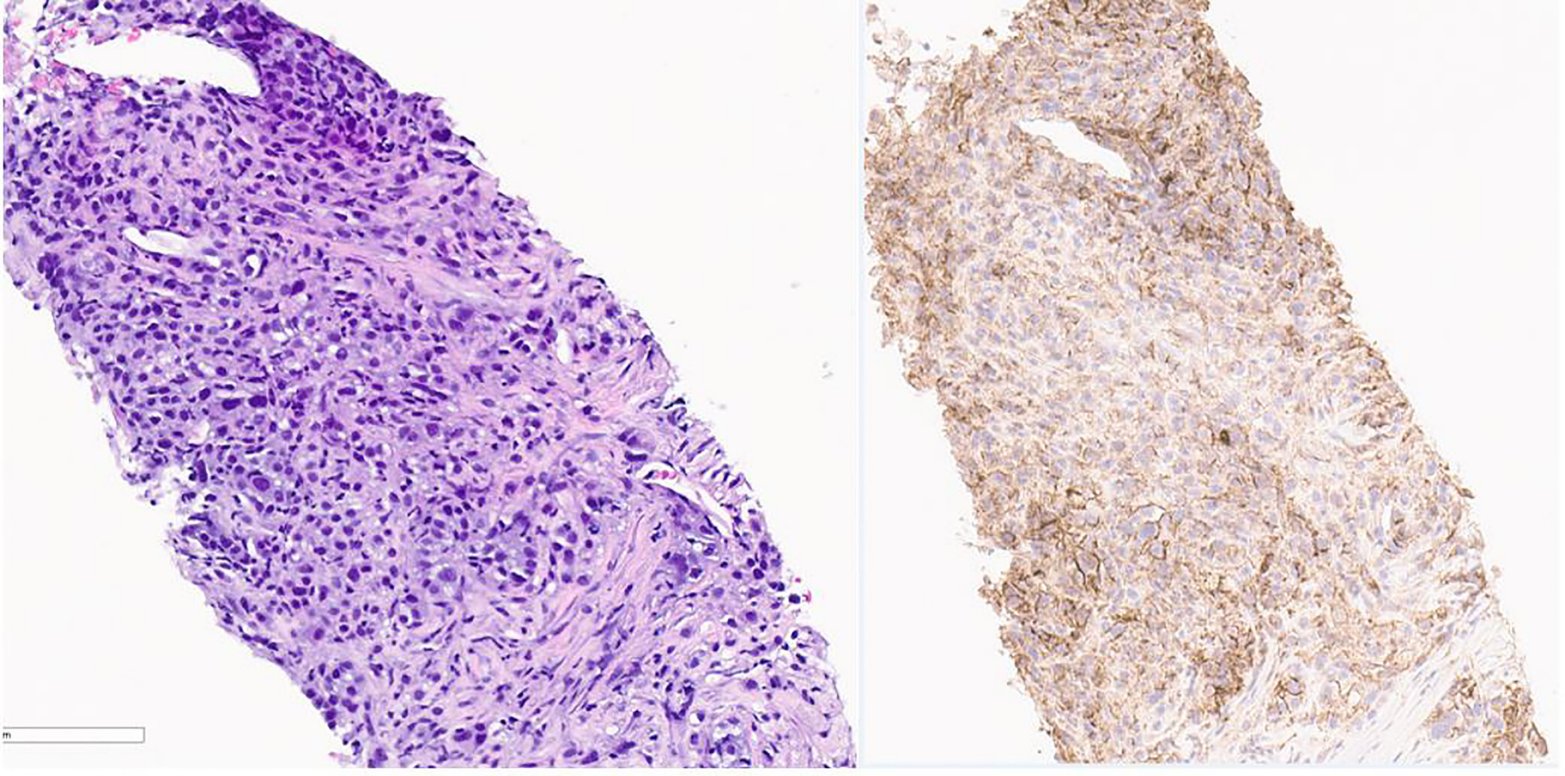
Pathology image of lung nodule biopsy showing PD-L-1 positivity. On the left is Hematoxylin & Eosin stain and on the right is PD L-1 immunohistochemistry stain showing PD-L-1 positivity. Pictures taken at 20x.

About 5 months after diagnosis of initial stage I B2 cervical cancer, she was seen in the emergency department with fevers and cough. CT chest with contrast done showed multiple pulmonary parenchymal nodules of varying sizes throughout the lungs consistent with diffuse pulmonary metastasis, and mediastinal and hilar adenopathy (**Figure 2**). She was started on treatment with three chemotherapy agents, i.e., cisplatin, paclitaxel, and bevacizumab, at 50% dose considering her delayed bone marrow recovery from pelvic radiation therapy. As she required frequent blood transfusions for anemia and had severe hypomagnesemia requiring frequent replacements, cisplatin was substituted for carboplatin during the 2nd cycle of chemotherapy. She could not tolerate the third cycle of chemotherapy due to severely compromised bone marrow and borderline kidney function. So systemic chemotherapy had to be discontinued considering her poor bone marrow reserve and associated nephrotoxicity. Her lung nodules biopsy came back 100% PD-L1 positive, so she was started on pembrolizumab only without additional chemotherapy, and imaging done a month later showed an interval decrease in the size of lung nodules (**Figure 3**).

A total of 4 months after starting on pembrolizumab, she had complaints of severe fatigue more than usual without complaints of palpitations, heat intolerance, and sweats. Blood work showed low thyroid-stimulating hormone (TSH) at 0.02. Prior to the initiation of pembrolizumab, TSH was within normal limits. Pembrolizumab can cause thyroid dysfunction and adrenal insufficiency, so cortisol was also checked; 8 a.m. cortisol came back normal at 6.4, so hypophysitis was ruled out. She was diagnosed with pembrolizumab-induced hyperthyroidism. Endocrinology was consulted, and she was started on propylthiouracil with a very good response. Her TSH started increasing, so propylthiouracil was discontinued. A decision was made to continue with pembrolizumab, as she had a very good response to treatment. Unfortunately, about 10 days after stopping propylthiouracil even before the next blood work, the patient was admitted to the intensive care unit with altered mental status and sinus bradycardia. She was intubated due to acute respiratory failure with hypercapnia secondary to myxedema coma and required pressor support with norepinephrine. She was treated with intravenous hydrocortisone and levothyroxine. Twenty-four hours later she was extubated, and 2 days later, she was discharged home with levothyroxine 100 μg daily. About 15 days later, there was a significant improvement in TSH to 25.74 from TSH of 200 at the time of presentation to the emergency department, so she was restarted on Keytruda. Follow-up imaging 2 months later to monitor response to Keytruda showed continued improvement in lung metastasis.

**Figure 4:**
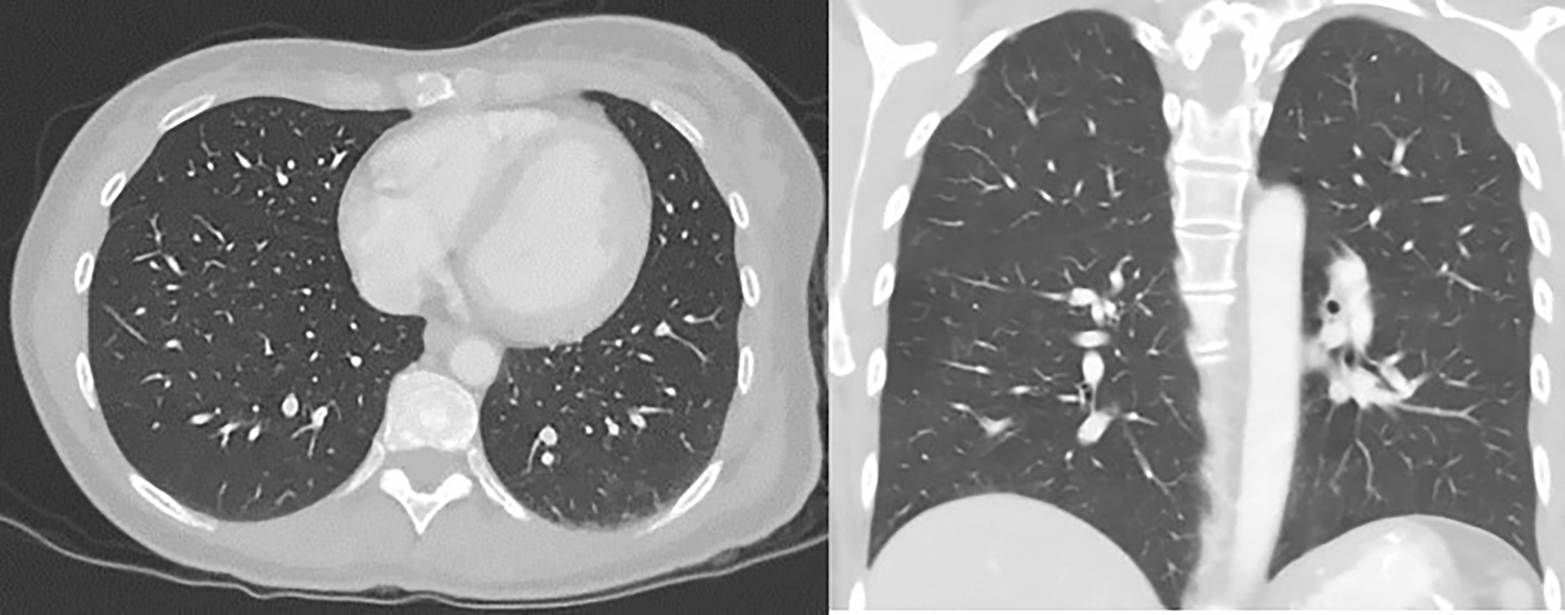
CT chest/abdomen with contrast after starting pembrolizumab showing a decrease in the size of lung metastasis.

**Figure 5:**
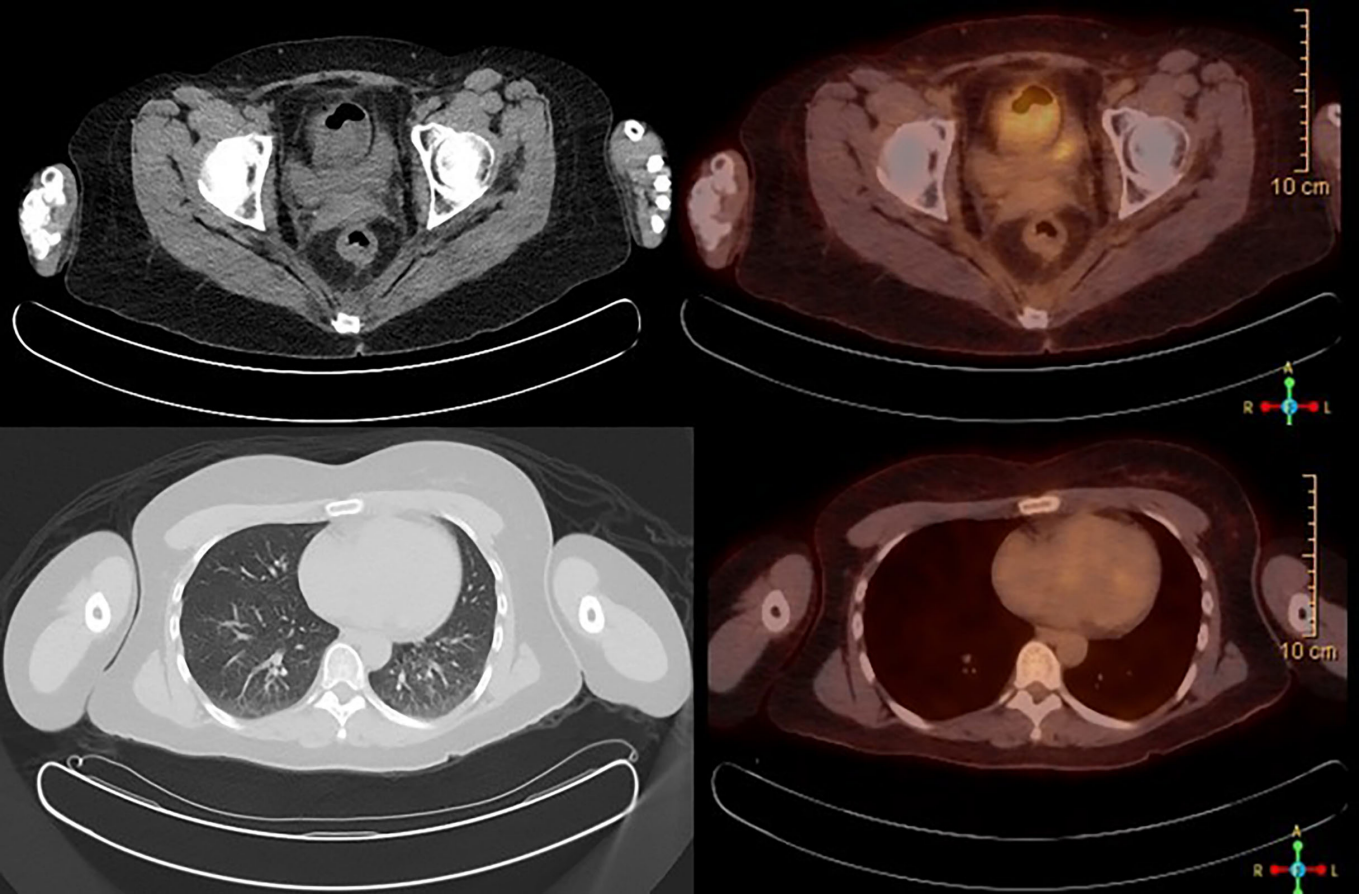
PET/CT scan showing no abnormal FDG avidity is seen in cervix, uterus, or lungs. No pulmonary nodules or metabolically active lymph nodes are identified in the mediastinum, axilla, abdomen, and pelvis. No axillary or supraclavicular lymphadenopathy.

Throughout the course of treatment, CT chest/abdomen/pelvis with contrast was repeated every 3 to 6 months, and PET skull base to mid-thigh with CT initial was repeated every 4 to 6 months to monitor the response to treatment. She showed consistent improvement in lung metastases (**Figure 4**). Pembrolizumab was continued every 3 weeks since diagnosis with some diarrhea as a side effect was well controlled with Imodium. Hypothyroidism was also well controlled with the help of levothyroxine. As of today, she continues to receive pembrolizumab every 3 weeks for the past 18 months. She finished 44 treatments with pembrolizumab till now. The last PET/CT in late 2021 showed no metabolically active cancer (**Figure 5**). She is doing clinically very well and is continuing to work full-time. She is currently completely asymptomatic without any pulmonary or systemic symptoms.

## Discussion

Cervical cancer is still the most common cancer detected in women worldwide. About 30 to 40 years ago, it was the most diagnosed gynecological cancer (8). The incidence has decreased in recent decades in developed countries due to screening and widespread HPV vaccinations (9, 10). However, it is still a common gynecological cancer in developing countries due to lack of resources and poor sanitation. Cervical cancer is caused by high-risk subtypes of HPVs. The common risk factors for this cancer are early sexual debut, multiple sexual partners, history of sexually transmitted infections, and tobacco abuse. It usually presents with symptoms of postcoital bleeding (11). Like every other cancer, it is divided into four stages depending on invasion to adjacent and distant organs. Treatment of cervical cancer is based on the stage at the time of diagnosis and the patient’s wish to preserve fertility. The relapse rate for cervical cancer is between 11% and 22% in the International Federation of Gynecology and Obstetrics (FIGO) stages 1B-IIA and 28%–64% in FIGO stages IIB-IVA. The persistence and local recurrence of cervical cancer often exceed the distant metastasis rate with increased pelvic tumor burden (12). Stage IV cervical cancer is a cancer that spreads to distant organs or it involves adjacent organs including the mucosa of the bladder or rectum (13). The overall survival rate of stage IV cervical cancer is only 17% compared to 85% in localized cervical cancer even with aggressive chemotherapy (14).

Treatment for metastatic cervical cancer is cisplatin-based chemotherapy, but according to research, no statistical benefit in overall survival was noted with platinum-based therapy (15). Its use is also limited due to significant toxicity and subsequent resistance to therapy (16). Bevacizumab is a monoclonal antibody with antiangiogenic properties and is also approved for metastatic cervical cancer. Adding bevacizumab to platinum-based chemotherapy in metastatic cervical cancer patients improved survival by 3.4 months, but it is associated with an increased risk for thromboembolism, fistulas, and a rare risk of bowel perforation (4). ICIs with chemotherapy or as a single agent raised a new hope in metastatic cancer treatments, as they are associated with improved overall survival rate and disease-free progression rates. Since the approval of ICIs by the Food and Drug Administration (FDA), their use has been incorporated in the treatment of about 19 different cancer types. There has been an increase in survival rates with the addition of ICIs in the treatment plan of patients with metastatic cancers including lung, cervix, colon, breast, esophagus, malignant melanomas, classical Hodgkin’s lymphoma, and other cancers (17).

PD-1, also called CD 279, was identified for the first time in 1992. CD 279 is expressed in activated cells including CD4 T, CD8 T, B cell, NK T cell, and CD4 CD8 negative T cells. It is a T-cell coinhibitory receptor, and its expression is increased in apoptotic cells, tumor cells, and virus infections (18, 19). The binding of PD-L1, the ligand for the PD-1 receptor, activates the PD-1/PD-L1 pathway leading to inhibition of T-cell activity (19, 20). The main role is to protect against excessive inflammatory responses and autoimmunity by increasing apoptosis in activated T cells (21). This mechanism also helps cancer cells escape our immune system leading to decreased antitumor response and progression of malignancies (22, 23). PD-1 and PD-L-I expression is upregulated in HPV-associated cervical tumor cells (24). Synthetic anti-PD-1 antibodies including pembrolizumab, nivolumab, and cemiplimab block this PD-1/PD-L-1 pathway leading to an improved immune response against tumor cells (25).

Pembrolizumab is a humanized IgG4 antibody against the PD-1 receptor accepted by the FDA. It was approved in 2014 for melanoma and non-small cell lung cancer by the FDA for the first time (26).

The treatment of stage IV cervical cancer is usually non-surgical with chemoradiotherapy, as combined radical surgery with chemoradiotherapy is associated with significant adverse events. Cisplatin-based chemotherapy with angiogenesis inhibitor bevacizumab has been the treatment for stage IV cervical cancer for many years. KEYNOTE-158 trial showed impressive results with pembrolizumab in previously treated advanced PD-L1-positive cervical cancer patients (27). Based on the results of the KEYNOTE-158 trial, in the year 2018, FDA granted accelerated approval of pembrolizumab use as a single agent for patients with metastatic cervical cancer with disease progression on or after receiving chemotherapy (28). It was the first immunotherapy agent approved for advanced gynecological cancer. EMPOWER-Cervical 1 trial compared anti-PD-1 cemiplimab with investigator choice (IC) single-agent chemotherapy in patients who progressed despite first-line platinum-based treatment. This landmark trial showed promising improvement in the overall survival of patients treated with immunotherapy compared to single-agent chemotherapy in patients regardless of their PD-1 status (29).

On October 13, 2021, pembrolizumab is accepted by the FDA as a first-line agent for use in combination with chemotherapy with or without bevacizumab in PD-L1-positive metastatic tumors based on the KEYNOTE-826 trial (6). Pembrolizumab now has regular approval by the FDA as a single agent for metastatic cervical cancer PD-L1 tumor-positive patients with disease progression on or after chemotherapy based on confirmatory data from the KEYNOTE-826 trial (4, 6, 30). The dose of pembrolizumab for cervical cancer is 200 mg 30-min infusion every 3 weeks or 400 mg 30-min infusion every 6 weeks until disease progression, until unacceptable toxicity, or up to 24 months (17). PD-L1-positive cervical cancer is a tumor with PD-L1 expression more than or equal to 1% in tumor cells. It is mostly used in combination with cisplatin-based chemotherapy, but as a single agent, the response rate remains low (31). Response to ICIs is determined by PD-L1 biomarker expression levels on tumor cells using *in vitro* immunohistochemistry assays. The assay shows the percentage of PD-L1-positive tumor cells compared to the viable cells in the sample (32). ICIs improve progression-free survival and overall survival in PD-L1-positive patients. Research shows that ICIs can also benefit PD-L1 negative patients with response rates between 11% and 20%. While the biomarker assay also identifies other PD-L1-positive cells including lymphocytes and macrophages, it has been shown that increased lymphocyte infiltration is associated with improved survival in many cancers. Increased CD-8+ T cells were noted in pretreatment melanoma cells in responders to pembrolizumab (33).

Cervical cancer was once the most common gynecological cancer even in developed countries. The incidence has decreased in the past few decades due to effective screening methods and HPV vaccinations. It remains an important cause of death in developing countries (34). It has a 75% to 85% overall 5-year survival rate if detected early (35). However, the overall 5-year survival rate is very low in patients with recurrent, persistent, or metastatic cancer (36). Our patient had metastatic cervical cancer to the lungs, and she could not tolerate cisplatin-based chemotherapy and bevacizumab, so she was treated with pembrolizumab as a single agent. She was 100% PD-L1-positive and responded very well to the immunotherapy for more than 2 years. Her last PET scan showed no FDG activity in lung nodules and cervix. We clearly understand the limitations of this case report. We treated only one young patient, and she could tolerate the immunotherapy without any toxicity. We also know she could not tolerate the standard chemotherapy, so we tried alternate immunotherapy, as we did not have any other option. To summarize, treating any metastatic cancer can be very challenging not only to the physicians but also to the patients. The treatment is physically and emotionally draining for the patients. Most patients are afraid not only about the side effects but also about their quality of life after starting the treatment for metastatic cancers. Immunotherapy increased expectations among oncologists about better treatment options for their patients. ICI as a monotherapy worked well in our patients, showing that it can possibly be used as a single agent in patients who cannot tolerate a combination of platinum-based chemotherapy and bevacizumab. The good outcome in our patients probably warrants further research on the use of immunotherapy as a single agent in patients with metastatic cervical cancer.

## Data Availability Statement

The original contributions presented in the study are included in the article/supplementary material. Further inquiries can be directed to the corresponding author.

## Author Contributions

MA wrote the case report under the guidance of MG. MG is the medical oncologist who is treating this patient. RS is the radiologist who added appropriate images pertinent to the text. All authors were involved in making appropriate changes as needed and approved the final case report.

## Conflict of Interest

The authors declare that the research was conducted in the absence of any commercial or financial relationships that could be construed as a potential conflict of interest.

## Publisher’s Note

All claims expressed in this article are solely those of the authors and do not necessarily represent those of their affiliated organizations, or those of the publisher, the editors and the reviewers. Any product that may be evaluated in this article, or claim that may be made by its manufacturer, is not guaranteed or endorsed by the publisher.

## Abbreviations

PD-1, programmed cell death protein-1; PD-L1, programmed cell death ligand-1; OB/GYN, obstetrician–gynecologist; ASCUS, atypical cells of undetermined significance; HPV, human papillomavirus; TSH, thyroid-stimulating hormone; PET, positron emission tomography; FDG, fluorodeoxyglucose; ICIs, immune checkpoint inhibitors; CD, cluster differentiation; B cell, mature in bone marrow, so B cell; NK cell, natural killer cell; T cell, final stage of development in thymus, so T cell; IgG, immunoglobulin G; FDA, Food and Drug Administration.
